# The Longitudinal Relationship Between Intrinsic Motivation, Grade Motivation and Prosocial Behavior in Chinese Adolescents: A Cross-Lagged Panel Study

**DOI:** 10.3390/bs16040563

**Published:** 2026-04-09

**Authors:** Xihao Niu, Han Liu, Kun Yan

**Affiliations:** 1 School of Education, Tsinghua University, Beijing 100084, China; niuxihao99@163.com; 2 School of Education, Minzu University of China, Beijing 100081, China; 24301640@muc.edu.cn

**Keywords:** intrinsic motivation, grade motivation, prosocial behavior, relational prosocial behavior, practical prosocial behavior, Chinese adolescents

## Abstract

Prosocial behavior is crucial for the development of adolescents. Prosocial behavior requires strong internal motivation to drive it, but the prospective association between the two is still unclear. In addition, there is still a gap in the relationship between grade motivation as a unique external motivation and prosocial behavior. To explore the prospective associations between them, we conducted two follow-up surveys (six months apart) on 862 Chinese adolescents (423 boys and 439 girls) to collect data on intrinsic motivation, grade motivation and prosocial behavior (relational prosocial behavior and practical prosocial behavior). The cross-lagged panel study results revealed that intrinsic motivation at T1 was positively associated with relational and practical prosocial behavior at T2. In contrast, grade motivation at T1 was not significantly associated with later prosocial behavior, and prosocial behavior at T1 was not significantly associated with later intrinsic motivation or grade motivation. These findings indicate that intrinsic motivation may be a potential antecedent for prosocial behavior in Chinese adolescents later in life, but the observed associations should be cautiously interpreted.

## 1. Introduction

Adolescence is a critical developmental period for the rapid restructuring of adolescents’ social functioning and peer relationships (Fu et al., 2024). Successfully navigating classroom peer interactions, such as collaborating with classmates, offering help, and responding to others’ needs, is closely related to adolescents’ social adaptation, sense of belonging, and the quality of their interpersonal relationships. Prosocial behavior, as a behavior that benefits others and promotes positive social relationships, is an important behavioral indicator of adolescents’ adaptive development (Crone & Achterberg, 2022), especially in the everyday school environment where most peer interactions occur. In the classroom, prosocial behaviors (such as helping, sharing, and comforting) can foster trust and reciprocity, reduce interpersonal conflict, and help create a more supportive peer atmosphere (Zhao et al., 2025), thereby promoting adolescents’ social integration and role development. Today, how to cultivate prosocial behavior in adolescents has become an important issue.

So far, scholars have explored various factors that may affect prosocial behavior, including intrinsic motivation. As a common form of motivation (Ryan & Deci, 2000a), intrinsic motivation can enhance an individual’s empathy and enable him to engage in activities that benefit others (Oh & Roh, 2022). Similarly, the development of prosocial behavior can enable individuals to meet their interpersonal needs while strengthening their intrinsic motivation (Righetti et al., 2022; Zou et al., 2024). Furthermore, in China, academic performance is still the main evaluation criterion for educational quality, and Chinese educators and parents generally attach more importance to education and students’ performance (Guo et al., 2018). Therefore, in this environment, coupled with the values of diligence instilled by parents, adolescents are eager to get good grades and realize their lofty ideals and ambitions (Jerrim, 2015). However, there is a lack of research on what kind of social behavior this motivation to get good grades will cause adolescents to have, and it is also unknown what impact prosocial behavior will have on this motivation. Although relevant academic performance research has provided some evidence (Li et al., 2023), the specific relationship between grade motivation and prosocial behavior still needs to be systematically demonstrated.

Although relevant studies have confirmed the influence of intrinsic motivation and grade motivation on adolescent prosocial behavior from a positive or indirect perspective, we urgently need to deepen our understanding of how these factors interact over time. Previous studies often rely on cross-sectional research methods, mainly examining the unidirectional relationship between these variables, and lack the dynamic interaction relationship between them. In addition, there is a significant gap in direct research on grade motivation. Therefore, to address these issues, this study examined the temporal sequence of intrinsic motivation, grade motivation, and prosocial behavior in a sample of Chinese adolescents across two time periods. Given the two-stage design, our aim was to examine prospective cross-lag associations, rather than changes within the developmental process or within individuals.

### 1.1. Self-Determination Theory and Prosocial Behavior

Self-determination theory (SDT) was proposed by Deci and Ryan (1980), which provides a theoretical framework for explaining the underlying mechanisms behind prosocial behavior (Kındap-Tepe & Aktaş, 2021). SDT posits that individuals have three psychological needs: autonomy (ownership and control over their own behavior and decisions), competence (successfully completing challenges and tasks), and relatedness (establishing close and meaningful relationships with others) (Ryan & Deci, 2020). When adolescents have these three needs met in their educational process and are motivated, they are more likely to actively engage in prosocial behavior (Deci & Ryan, 2000). At the same time, SDT also posits that higher motivation is associated with numerous positive outcomes, including participation in more social behaviors (Peetz & Milyavskaya, 2021). In addition, SDT divides motivation into intrinsic motivation and extrinsic motivation. When an individual’s prosocial behavior is motivated by intrinsic or extrinsic motivation, the quality of the behavior will vary greatly (Kındap-Tepe & Aktaş, 2021). In other words, intrinsic motivation and extrinsic motivation will have different effects on prosocial behavior.

Prosocial behavior refers to behavior that individuals voluntarily perform to benefit others or society. It is characterized by kindness, compassion, and concern for the rights, feelings, and well-being of others (Barton et al., 2020). As a key element in maintaining social connections and promoting individual development, prosocial behavior is of great significance in the process of individual socialization. It not only shapes individual character, but also affects group atmosphere and social harmony (Van Der Graaff et al., 2018). In recent years, with the in-depth study of prosocial behavior, the multidimensionality of prosocial behavior has been increasingly emphasized (Ju et al., 2025). Kındap-Tepe and Aktaş (2021) specifically categorized prosocial behavior into compliant and anonymous prosocial behavior. Therefore, in this study, based on the research of Warden et al. (2003), we further divided prosocial behavior into relational prosocial behavior and practical prosocial behavior. Combining with their research, we defined relational prosocial behavior as behavior that strengthens interpersonal bonds through emotional support (such as “caring for, comforting, and accepting classmates”) and meets the relationship needs of individuals; and defined practical prosocial behavior as behavior that solves practical problems by providing specific, instrumental helping behaviors (such as “helping up injured classmates”), which often relies more on the individual’s skills to execute.

### 1.2. Intrinsic Motivation and Prosocial Behavior

Intrinsic motivation refers to an individual engaging in an activity for their own intrinsic satisfaction, rather than for some detachable results. When an individual is driven by intrinsic motivation, he or she will take action for the pleasure or challenge inherent in the activity, rather than because of external stimulation, pressure or reward (Ryan & Deci, 2000b). In other words, people with intrinsic motivation will gain rewards and pleasure from the performance or activity itself (Teppo et al., 2021). Furthermore, the positive emotions experienced by individuals with high intrinsic motivation at work may enhance their empathy tendencies, making them feel closer to others (Oh & Roh, 2022).

Intrinsic motivation predicts prosocial behavior. This is because individuals with higher intrinsic motivation are more likely to value intrinsic motivation and associate it with higher moral values (Kwon & Sonday, 2024). When individuals moralize intrinsic motivation, they perceive those with higher intrinsic motivation as more moral, and thus exhibit more prosocial behavior toward them (Kwon et al., 2023). Related empirical studies have also expressed the same view. For example, Jin et al. (2024) found that Chinese medical students have intrinsic motivation to engage in prosocial behaviors that are consistent with their core values, thereby reducing professional burnout and benefiting others. Oh and Roh (2022) showed from the perspective of individual work that the intrinsic motivation of individual work activities will cultivate empathy, and empathy will promote prosocial behavior, thereby bringing more social support. Similarly, a study on young children showed that prosocial behavior stems from multiple motivations, and one of the prosocial behaviors, such as altruism, is driven by intrinsic motivation (Spinrad & Gal, 2018). This suggests that adolescents’ intrinsic motivation can drive them to engage in prosocial acts that benefit others and society.

Prosocial behavior predicts intrinsic motivation. Because when prosocial behaviors are carried out autonomously and meet the needs of interpersonal relationships, they are more likely to enhance intrinsic motivation and happiness (Chen, 2025). Related research has also verified this view from the opposite side. For example, Lee et al. (2019) found that introducing external rewards for prosocial behavior sometimes weakens intrinsic motivation, especially when the rewards are perceived control rather than support, which indirectly indicates that prosocial behavior enhances the intrinsic motivation of individuals. Kitzrow (1998) divided the development of prosocial behavior into six stages. The later development stage is characterized by a decrease in external motivation and an increase in internal motivation. In other words, as prosocial behavior develops, the internal motivation of individuals continues to develop. At the same time, this view is also confirmed by the study of autonomy. Gagné (2003) found that autonomy is closely related to prosocial behavior. Autonomy refers to the ability of individuals to independently decide what is most beneficial to themselves, and satisfaction with autonomy contributes to students’ intrinsic motivation (Ryan et al., 2021).

### 1.3. Grade Motivation and Prosocial Behavior

Grades are important short-term goals. They are a measure of academic success (York et al., 2015). In this study, grade motivation refers to adolescents’ desire to achieve good grades and excel in academic assessment situations (Mega et al., 2014). This concept should not be understood as a universal measure of all forms of extrinsic motivation, nor should it be considered a complete representation of achievement-oriented motivation. Instead, it captures a performance-centered, controlled academic motivation, at its core being the emphasis on good academic outcomes. Drawing on SDT (Ryan & Deci, 2000b), grade motivation can be initially positioned as closer to controlled regulation than intrinsic motivation, because it focuses more on externally valued results rather than the intrinsic pleasure of learning itself. Furthermore, since the pursuit of good grades may also involve concerns about performance and social comparison, this concept is conceptually similar to performance-oriented motivation, although it is not equivalent to the broader performance-oriented framework.

Grade motivation may predict prosocial behavior. So far, there are almost no studies on the relationship between grade motivation and prosocial behavior, but some related studies provided a solid foundation for this study. For example, Guo et al. (2018) showed from the perspective of academic performance that most Chinese parents value education and are eager for their children to achieve good academic results. As a result, students were forced to devote almost all their time to learning activities, while social participation is seriously underestimated (Parmar et al., 2004), which is not conducive to the development of students’ prosocial behavior. Also, from the perspective of academic performance, Li et al. (2023) found that prosocial behavior is related to positive academic outcomes (academic self-efficacy or higher grades point average). In other words, better grades can help adolescents increase prosocial behavior. In addition, some studies have shown from the perspective of learning methods that students who adopt cooperative learning methods to achieve their own goals (such as reducing aggression or getting good grades) are often more likely to engage in prosocial behavior when interacting with peers and reduce individualistic tendencies (Choi et al., 2011).

Prosocial behavior may also predict grade motivation. Similarly, there is no direct research to explore the impact of prosocial behavior on grade motivation, and most studies have explored the impact of prosocial behavior on academic performance. However, this part of the research can also provide solid evidence for our research. For example, Gerbino et al. (2018) found that adolescent prosocial behavior can not only predict intelligence and personality traits, but also predict good academic performance. Because study has shown that prosocial behavior can improve academic performance by building a positive interpersonal network in the classroom (Rehman et al., 2024; Wentzel, 1993). A recent longitudinal study also indicated that prosocial behavior has a positive impact on academic performance, but not directly; rather, it exerts an indirect influence through students’ self-efficacy (Cruz et al., 2025). Good academic performance is the result form of grade motivation, so we have reason to believe that adolescents’ prosocial behavior exerts a positive influence on grade motivation.

### 1.4. Present Study

Existing studies have shown that there may be a reciprocal relationship between intrinsic motivation, grade motivation and prosocial behavior. However, previous studies have examined the relationship between motivation and prosocial behavior solely from a cross-sectional or holistic perspective, but have not explored the longitudinal relationship between the two, nor have they explored the internal structural relationship between motivation and prosocial behavior in detail. In order to overcome this limitation in research methods and research content, this study adopted a longitudinal research design and conducted two consecutive follow-up surveys on Chinese adolescents, aiming to clarify the prospective associations in the relationships between Chinese adolescents’ intrinsic motivation, grade motivation, and prosocial behavior, and paid special attention to the relationship between two motivations and two specific types of prosocial behaviors and proposed four main hypotheses and eight sub-hypotheses:

**Hypothesis** **1.** *Chinese adolescents’ intrinsic motivation at T1 will be positively associated with prosocial behavior at T2.*

**Hypothesis** **1a.** *Chinese adolescents’ intrinsic motivation at T1 will be positively associated with relational prosocial behavior in prosocial behavior at T2.*

**Hypothesis** **1b.** *Chinese adolescents’ intrinsic motivation at T1 will be positively associated with practical prosocial behavior in prosocial behavior at T2.*

**Hypothesis** **2.** *Chinese adolescents’ grade motivation at T1 will be positively associated with prosocial behavior at T2.*

**Hypothesis** **2a.** *Chinese adolescents’ grade motivation at T1 will be positively associated with relational prosocial behavior in prosocial behavior at T2.*

**Hypothesis** **2b.** *Chinese adolescents’ grade motivation at T1 will be positively associated with practical prosocial behavior in prosocial behavior at T2.*

**Hypothesis** **3.** *Chinese adolescents’ prosocial behavior at T1 will be positively associated with intrinsic motivation at T2.*

**Hypothesis** **3a.** *Chinese adolescents’ relational prosocial behavior in prosocial behavior at T1 will be positively associated with intrinsic motivation at T2.*

**Hypothesis** **3b.** *Chinese adolescents’ practical prosocial behavior in prosocial behavior at T1 will be positively associated with intrinsic motivation at T2.*

**Hypothesis** **4.** *Chinese adolescents’ prosocial behavior at T1 will be positively associated with grade motivation at T2.*

**Hypothesis** **4a.** *Chinese adolescents’ relational prosocial behavior in prosocial behavior at T1 will be positively associated with grade motivation at T2.*

**Hypothesis** **4b.** *Chinese adolescents’ practical prosocial behavior in prosocial behavior at T1 will be positively associated with grade motivation at T2.*

## 2. Materials and Methods

### 2.1. Procedure and Participants

This study used a convenient sampling method to recruit adolescent volunteers from four junior high schools in four Chinese provinces (Yunnan, Hubei, Jiangsu and Henan). All participants and their parents or legal guardians signed written informed consent forms. Participation was entirely voluntary, and respondents were informed of their right to withdraw at any time without penalty. Furthermore, this study was approved by the administrative departments of the participating schools. Data were collected via offline questionnaires at two time points in 2025. At each participating school, we randomly selected students from a list. For the survey, we grouped the selected students together in designated classrooms and had them complete questionnaires simultaneously. Importantly, we did not select students from entire classes (i.e., we did not survey them in complete groups within their original classes). Therefore, while students are still nested within the school, the potential clustering effect due to shared instruction at the class level may be reduced. However, potential school-level clustering may still exist. During data collection, dedicated researchers were present in the classrooms to provide procedural guidance and ensure that participants completed the questionnaires independently. Staff did not observe or evaluate any individual’s responses. Although researchers were present to supervise data collection, participants were told that their responses were anonymous and used solely for research purposes. However, the possibility of societal expectation bias could not be completely ruled out. After the answers were completed, they were collected and entered. The evaluation interval was 6 months. All information provided by participants was kept strictly confidential. This study was approved by the research ethics committee of the author’s unit.

In May 2025, this study invited Chinese adolescents to participate in the survey. A total of 1089 adolescents agreed to participate and completed the full questionnaire at Time 1 (T1). 862 students completed the second point (Time 2, T2) data collection in November 2025. The final sample retained data from 862 participants who participated in both assessments, including 423 male participants (49.1%) and 439 female participants (50.9%). All participants were in grades 7–9, aged 12–15, with an average age of 13.5.

To examine potential attrition effects, we conducted independent-sample t-tests on the four variables between attrited and retained participants. As shown in Table 1, the t-values ranged from −1.716 to 0.757 (*p* > 0.05), indicating that the sample attrition was random. We then conducted the Missing Completely at Random test. This test primarily examined wave-level attrition. The result (χ^2^ = 17.728, df = 14, *p* = 0.219) showed that in the current study, the data were completely missing at random and the loss did not affect the representativeness of the final sample.

### 2.2. Measures

This study primarily employed two scales: Glynn et al. (2011)’s Scientific Motivation Scale and Warden et al. (2003)’s Prosocial Behavior Scale. Regarding motivation, we focused specifically on intrinsic and grade motivation in general learning contexts among adolescents. Therefore, instead of using the full Scientific Motivation Scale, we selected the sub-dimensions most relevant to our research questions. Since the scale developed by Glynn et al. (2011) was initially designed for science learning contexts, and this study examines general learning motivation in adolescents rather than motivation in a specific science domain, we systematically replaced subject-specific terms (e.g., “science”) with domain-general terms (e.g., “learning”) while preserving the original meaning of each item. Therefore, the intrinsic and grade motivation in this study should be interpreted as indicators of general learning motivation, not motivation in a specific science domain.

To reduce the burden on participants in the two-stage design and minimize sample attrition, we employed a concise three-item measurement tool for each motivation concept. Item selection followed three principles: (a) conceptual consistency with the original construct definition; (b) suitability for the developmental level and language expression of Chinese junior high school students; and (c) suitability for repeated measurement in both stages. Therefore, we prioritized items that were clearly worded, highly representative of each construct, and easy to administer longitudinally.

For prosocial behavior, we retained the relational and practical dimensions of the scale from Warden et al. (2003), as these two dimensions are theoretically crucial for our goal of examining the internal structure of adolescent prosocial behavior. Furthermore, since this is the first time this scale has been used in a Chinese cultural context, we conducted meticulous translation and linguistic adjustments. Specifically, two research assistants first translated the original items into Chinese, followed by bilingual proofreading by a native English speaker and a Chinese English teacher to improve semantic clarity and conceptual consistency. Items were shortened only when necessary to reduce participant burden, while maintaining the conceptual coverage of each construct.

#### 2.2.1. Intrinsic Motivation

The intrinsic motivation of adolescents was mainly assessed using the Intrinsic Motivation Scale (Glynn et al., 2011). The scale contains three questions, such as “Learning makes me feel that life is meaningful.” Responses were rated on a 5-point Likert scale (1 = “completely disagree”, 5 = “completely agree”), with higher scores indicating stronger intrinsic motivation in adolescents. The Cronbach’s alpha coefficients of the scale at two time points (T1 and T2) were 0.876 and 0.783, and its composite reliability was 0.880 and 0.806, respectively.

#### 2.2.2. Grade Motivation

The grade motivation of adolescents was mainly assessed using the Grade Motivation Scale (Glynn et al., 2011). The scale contains three questions, such as “Good grades are very important to me”. Responses were rated on a 5-point Likert scale (1 = “completely disagree”, 5 = “completely agree”), with higher scores indicating stronger grade motivation in adolescents. The Cronbach’s alpha coefficients of the scale at two time points (T1 and T2) were 0.797 and 0.813, and its composite reliability was 0.799 and 0.827, respectively.

#### 2.2.3. Prosocial Behavior

The prosocial behavior of adolescents was mainly assessed using the Children’s Prosocial Behavior Scale (Warden et al., 2003). This scale contains two dimensions: relational prosocial behavior and practical prosocial behavior. The relational prosocial behavior scale contains 4 questions, such as “I will be friendly to sad classmates in the class.” Responses were rated on a 5-point Likert scale (1 = “completely disagree”, 5 = “completely agree”), with higher scores indicating stronger relational prosocial behavior in adolescents. The Cronbach’s alpha coefficient of this scale at the two time points (T1 and T2) was 0.713 and 0.750, and its composite reliability was 0.723 and 0.756, respectively. The practical prosocial behavior scale contains 4 questions, such as “I will help classmates who fall or are injured.” Responses were rated on a 5-point Likert scale (1 = “completely disagree”, 5 = “completely agree”), with higher scores indicating stronger practical prosocial behavior in adolescents. The Cronbach’s alpha coefficient of this scale at the two time points (T1 and T2) was 0.724 and 0.735, and its composite reliability was 0.753 and 0.705, respectively.

### 2.3. Data Analysis

This study mainly used two software packages, SPSS 27.0 and Mplus 8.3, for analysis. First, we used SPSS 27.0 to test the attrition rate, followed by descriptive statistics and correlation analysis. Second, we used Mplus 8.3 for confirmatory factor analysis (CFA) and longitudinal invariance testing. Finally, we used Mplus 8.3 to construct an observed-variable cross-lagged panel model (CLPM) for analysis.

The cross-lagged panel model was estimated in Mplus 8.3 using robust maximum likelihood estimation (MLR). All focal constructs (intrinsic motivation, grade motivation, relational prosocial behavior, and practical prosocial behavior) were operationalized as observed variables using scale means. Although individual questionnaire items were measured on Likert-type scales, scale means were treated as continuous indicators, which is common practice in structural equation modeling.

Missing data across the two waves were addressed via listwise deletion, such that only participants who completed both waves were included in the longitudinal analyses. The overall retention rate was 79.16%. Attrition analyses were conducted to assess potential systematic differences between retained and non-retained participants.

Furthermore, although participants were not surveyed within complete classes, they were still nested within schools, so school-level clustering effects may still exist. Because school identifiers are not preserved, we cannot estimate multilevel models or calculate the standard error of cluster robustness. Therefore, if the similarity among students within the same school is higher than expected under the independence assumption, the standard error of the cross-lag coefficients may be underestimated, which in turn may lead to overly lenient significance tests. This problem is particularly important when interpreting statistically significant cross-lag coefficients, as their values are small. Therefore, all inferences should be cautiously interpreted.

## 3. Results

### 3.1. Descriptive Statistics

First, the mean and standard deviation of each variable of the adolescents were tested. As shown in Table 2, adolescents’ intrinsic motivation, grade motivation, and prosocial behavior were all at the upper-middle level (M > 3) at both time points. Second, to assess changes in the mean scores of the variables over time, paired-samples t-tests were conducted, with results presented in Table 2. Significant differences were observed in adolescents’ intrinsic motivation, grade motivation, and prosocial behavior at T2 compared with T1. Average differences are reported in a descriptive manner; however, since scalar invariance has not yet been established, the observed average changes should be cautiously interpreted, as they may partially reflect changes in the item intercept or threshold rather than the true changes.

### 3.2. Correlation Analysis

As shown in Table 3, significant positive correlations were observed among Chinese adolescents’ intrinsic motivation, grade motivation, and prosocial behavior. This result lays a good data foundation for the further development of our research.

### 3.3. Evaluation of the Measurement Model

This study used CFA to assess the measurement characteristics of intrinsic motivation, grade motivation, relational prosocial behavior, and practical prosocial behavior at two time points. As shown in Table 4, all standardized factor loadings were statistically significant (*p* < 0.001). The standardized factor loadings for intrinsic motivation ranged from 0.782 to 0.930 at T1 and from 0.693 to 0.798 at T2, indicating high reliability and time stability. The factor loadings for grade motivation were also satisfactory, ranging from 0.587 to 0.894 at T1 and from 0.622 to 0.929 at T2, supporting an adequate representation of this construct across different time points. For relational prosocial behavior, most indicators showed moderate to strong factor loadings (0.491–0.717 at T1; 0.496–0.781 at T2). Although the factor loadings for RPB2 were relatively low (0.491 at T1, 0.496 at T2), these values still exceeded the minimum threshold of 0.40 that is typically recommended for item retention in psychological research (Cheung et al., 2024; Stevens, 2002). Given the item’s theoretical relevance to the construct domain and its significant consistency across both time points, it was retained to maintain content validity. Similarly, practicing prosocial behavior showed acceptable factor loadings at T1, ranging from 0.463 to 0.812, and at T2, from 0.415 to 0.773. While the factor loadings for PPB3 and PPB4 were relatively low (0.415–0.563), these values still met the minimum retention criteria for the established scale (Cheung et al., 2024; Stevens, 2002), especially given the priority given to theoretical coverage and longitudinal comparability. Removing these items would reduce the conceptual breadth of the constructs and impair longitudinal invariance.

In conclusion, the CFA results support the validity of the measurement model. All indicators have significant loadings on their respective underlying constructs, and the overall pattern of the loadings suggests that the construct has acceptable validity and measurement stability. At the same time, the relatively low loadings of some indicators cannot be ignored. This may indicate that these simplified measurement methods may have limited sensitivity in capturing the full variability of the underlying structure, especially in detecting subtle longitudinal variations. Therefore, the measurement results should be interpreted as acceptable but not optimal, and the observed correlations may be slightly conservative or weakened.

### 3.4. Longitudinal Measurement Invariance

Before constructing the observed-variable CLPM, we first need to conduct a longitudinal invariance test on adolescents’ intrinsic motivation, grade motivation, and prosocial behavior at two time points. As shown in Table 5, the changes in CFI and TLI are within acceptable ranges. Therefore, Configural model and Metric invariance between the two time points are supported. However, Scalar invariance is not confirmed. This suggests that factor loadings are sufficiently stable over time to support analyses of construct associations, but equal item intercepts cannot be assumed. Therefore, comparisons of observed or latent means at different time points should be cautiously interpreted and should not be considered the primary basis for substantive inferences in this study.

Since Scalar invariance has not yet been established, we do not interpret the difference in average levels between T1 and T2 as substantial evidence of developmental change. Therefore, the longitudinal analysis below focuses on cross-lag relationships between constructs rather than changes in mean scores over time.

### 3.5. Cross-Lagged Panel Analysis

Preliminary analyses revealed no significant gender differences in intrinsic motivation, relational prosocial behavior, and practical prosocial behavior (all *p* > 0.10). Although a statistically significant gender difference was observed in adolescents’ grade motivation (*p* = 0.028), the effect size was small (Cohen’s *d* = 0.15), indicating limited practical significance. Therefore, gender was not included as a control variable in the primary analysis.

**Figure 1:**
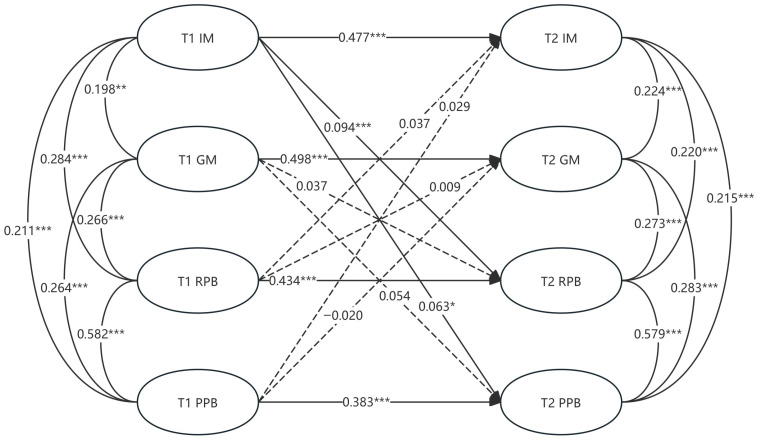
The cross-lagged panel model. * *p* < 0.05, ** *p* < 0.01, *** *p* < 0.001.

As illustrated in Figure 1, we observed significant autoregressive effects for all individual variables from T1 to T2 (0.383 ≤ β stability ≤ 0.498, *p* < 0.001). The most stable effect was observed for grade motivation (*β* = 0.498, *p* < 0.001). Furthermore, adolescents’ intrinsic motivation at T1 showed significant positive cross-lagged associations with relational prosocial behavior and practical prosocial behavior at T2. However, these coefficients are small and should be cautiously interpreted, especially since clustering effects at the school level cannot be modeled, and therefore the standard error may be underestimated. In contrast, grade motivation at T1 did not show significant cross-lagged associations with either type of prosocial behavior at T2, and prosocial behavior at T1 did not show significant cross-lagged associations with later intrinsic motivation or grade motivation.

Although Table 2 shows a moderate degree of correlation among some variables, the cross-lag path coefficients derived in Figure 1 represent more conservative estimates because they control for autoregressive effects and contemporaneous relationships. Therefore, differences between the correlation coefficients and path coefficients are expected.

## 4. Discussion

The results showed that intrinsic motivation at T1 was prospectively associated with both relational and practical prosocial behavior at T2 after accounting for autoregressive effects and within-wave correlations. However, these associations are weak and should be considered preliminary results, as it is impossible to model clustering effects at the school level, and therefore the standard error may be underestimated. By contrast, grade motivation at T1 was not significantly associated with later prosocial behavior, and prosocial behavior at T1 was not significantly associated with later intrinsic motivation or grade motivation. It is important to note that because these findings are based on two-wave CLPM, they should be interpreted as prospective associations rather than evidence of developmental, causal, or intra-individual directional effects.

### 4.1. Intrinsic Motivation and Prosocial Behavior

In this study, adolescents’ intrinsic motivation showed a significant prospective association with later prosocial behavior across the two-wave period. According to SDT, intrinsic motivation can provide psychological motivation for adolescent prosocial behavior by satisfying three basic psychological needs: autonomy, competence, and relatedness (Deci & Ryan, 2000). This may be because individuals whose basic psychological needs are satisfied experience less stress when helping others, thereby activating positive emotions that drive prosocial behavior and greater effort toward such acts (Peetz & Milyavskaya, 2021). At the same time, studies have shown that compared with the other two psychological needs, the satisfaction of relatedness is more important in promoting adolescent prosocial behavior (Miles & Upenieks, 2022). Because it helps to improve adolescents’ empathy and social skills, establish close relationships with others through social interactions, and then promote prosocial behavior (Oh & Roh, 2022). In other words, individuals with high intrinsic motivation are more likely to be able to acutely perceive others’ emotional needs in interpersonal interactions, experience empathy, and undergo faster internalization, thereby naturally exhibiting supportive behaviors. Therefore, when intrinsic motivation is high, it may promote adolescents’ prosocial behavior, strengthen and amplify the effect of prosocial behavior, and prosocial behavior driven by intrinsic motivation will also bring pleasure and sense of accomplishment by itself, forming a virtuous circle of “maintaining prosocial behavior”.

However, it is noteworthy that intrinsic motivation exhibits a relatively small path effect on relational and practical prosocial behaviors. This may be because intrinsic motivation primarily drives behaviors motivated by interest or self-satisfaction, while relational and practical prosocial behaviors rely more on emotional resonance, social norms, or external rewards, resulting in a relatively low motivation-behavior fit. Furthermore, the short-scale measurement used in this study to measure intrinsic motivation and prosocial behavior, along with the normative constraints within the school setting, may further weaken the direct linkage of intrinsic motivation. This result is consistent with self-determination theory (Ryan & Deci, 2000b) and related empirical research (Zhang & Huang, 2025), which suggests that intrinsic motivation typically has a weaker linkage on extroverted, other-oriented behaviors, and may exert its influence more through indirect pathways. In addition, since clustering effects at the school level cannot be accounted for, the statistical significance of these smaller coefficients should not be over-interpreted. These findings are best viewed as preliminary evidence of potential future associations rather than as reliable estimates of effect sizes.

From a practical perspective, the magnitude of these effects is relatively small, suggesting that intrinsic motivation alone may be unlikely to significantly alter adolescents’ prosocial behavior over time. In the everyday school environment, prosocial behavior is influenced by a variety of factors beyond individual motivation, such as peer interaction, classroom norms, and situational expectations. Therefore, although the observed effects are statistically detectable, they should be understood as weak contributions to a broader system of influences rather than strong predictive relationships. Furthermore, such small effect sizes are not uncommon in longitudinal cross-lag models. In this context, our findings are generally consistent with existing longitudinal studies (Carrizales et al., 2021), namely that cross-lag effects are typically small but still contribute to understanding developmental processes.

In contrast, the reason why prosocial behavior did not show a significant prospective association with later intrinsic motivation across the two-wave period may be due to the characteristics of Chinese social interaction. Because, within the collectivist cultural context (Chan & Lam, 2023), Chinese adolescents adhere to the concept of the Great Harmony. They believe that helping others is their nature and obligation, so it will not affect intrinsic motivation. Similarly, it may be because China is a society that values human relationships, where social interactions emphasize emotional support, friendship maintenance and other relation-oriented prosocial behaviors, which usually occur in established and familiar interpersonal relationships and are accompanied by strong expectations of reciprocity and social norms, such as caring obligations between friends. This makes the behavioral motivation more driven by external relationship maintenance rather than pure autonomous will. Importantly, conceptualizing prosocial behavior as a cultural obligation does not imply that such behavior is entirely independent of intrinsic motivation. On the contrary, intrinsic motivation may function as an internal psychological resource that enables adolescents to engage in prosocial behavior voluntarily and consistently. In other words, although fulfilling cultural obligation may not directly enhance intrinsic motivation, adolescents with higher levels of intrinsic motivation are more likely to enact prosocial behavior in a more autonomous, sustained, and positive manner.

### 4.2. Grade Motivation and Prosocial Behavior

In this study, adolescents’ grade motivation did not show a significant prospective association with later prosocial behavior across the two-wave period. This may be because, within China’s exam-oriented educational context (Zeng & Huang, 2021), adolescents face high parental expectations, and Chinese parents hope that their children can get a better life by getting good grades. Therefore, adolescents are eager to maintain the parent–child relationship with their parents by getting good grades, which may lead to some adolescents using the grade motivation as a powerful driving force to maintain the parent–child relationship. Secondly, it may also be because teenagers want to achieve better grades, especially better grades in the class, which has contributed to the unhealthy competitive atmosphere in the school, and this atmosphere may, in some cases, shift students’ attention toward self-focused academic outcomes (David et al., 2021). Therefore, China’s highly competitive academic environment may narrow the focus of grade motivation to personal ranking rather than collective welfare. Finally, some norms derived from Confucian educational traditions may continue to shape expectations regarding helping others and academic striving in contemporary Chinese school settings. So prosocial behavior may be more driven by Confucian obligation norms, emphasizing the cultivation of non-instrumental prosocial behavior, which is somewhat different from the grade motivation.

Similarly, adolescents’ prosocial behavior did not show a significant prospective association with later grade motivation across the two-wave period. Firstly, one possible interpretation may be that some adolescents may view certain forms of prosocial behavior as socially expected or normatively appropriate, thus it may not affect their grade motivation. Secondly, if adolescents devote a significant amount of time to prosocial behavior, this may reduce their study time, thereby diminishing their desire for good grades. (Fredricks, 2012). Given that executive functions and self-regulatory capacities are still developing during adolescence, adolescents may not yet be fully able to balance social engagement and academic demands (Best et al., 2009). At the same time, the expectations of important others, such as parents, teachers, and friends, may be more likely to motivate adolescents (Eccles & Wigfield, 2020). This is because when they believe in the potential of adolescents, this “positive prediction” is internalized into their self-belief, creating a “Pygmalion effect”, prompting adolescents to strive to achieve the excellent image in the eyes of others (Jussim & Harber, 2005). Therefore, we cannot assume that “being helpful equates to good grades”. This is because the motivations underlying prosocial behavior are typically intrinsic, altruistic, or relationship-oriented, stemming from a different psychological system than that of grade motivation. However, in the long run, actively participating in prosocial activities often shapes a more complete and socially responsible individual. These traits undoubtedly form a solid foundation for long-term academic success and life achievements.

### 4.3. Implications

Theoretically, our longitudinal model provides evidence that adolescents’ intrinsic motivation showed a significant prospective association with later prosocial behavior across the two-wave period, suggesting that adolescents’ autonomous helping motivation may be a significant antecedent for their subsequent prosocial behavior. However, the model does not support a two-way path between prosocial behavior and subsequent motivation, nor does it provide evidence of a cross-lag effect between grade-level motivation and prosocial behavior.

Practically, the findings suggest that efforts to promote adolescent prosocial behavior may benefit from their intrinsic motivation. Schools could explore to adopt autonomy-supportive practices (e.g., providing rational explanations, acknowledging students’ perspectives, and offering appropriate choices), foster respectful teacher-student relationships, and encourage voluntary mutual assistance among students. Families may consider to support adolescents’ autonomous motivation by maintaining warm, low-conflict interactions and avoiding harsh or hostile disciplinary approaches. However, it should be noted that these findings should be considered as preliminary guidance for intervention design, rather than final guidance, due to the small observed cross-lag coefficients and the inability to model clustering at the school level.

### 4.4. Limitations and Future Research

First, the longitudinal tracking of this study only conducted two time points of design collection. The limited number of measurement time points hinders the examination of temporal mediating effects between variables and potential circular causal relationships. Therefore, in further research, the number of measurements should be increased and the measurement time should be extended to make the research results more scientific and rigorous. Second, the current study only used questionnaires to investigate the relationship between motivation and prosocial behavior of Chinese adolescents. The research subjects were mainly concentrated on adolescents, and there was a lack of investigations on other groups, which may produce self-report bias. Therefore, in further research, it is necessary to combine multiple survey methods such as interviews and observations, and expand the research subjects to make the research results more accurate and reasonable. Third, because the present study collected data at only two time points, the analyses were limited to the traditional cross-lagged panel model. Although this model is useful for examining temporal associations between variables, it primarily captures between-individual stability rather than within-individual change. To address this limitation, future research will employ data collected across three or more time points and apply a random intercept cross-lagged panel model to disentangle stable between-person differences from dynamic within-person processes. Fourth, a six-month interval was established between T1 and T2. During this period, participants engaged in routine academic activities, including coursework assessments and examinations, which were likely to influence their grade motivation. Because academic events such as examinations and performance feedback occurred within the measurement interval, unmeasured contextual factors may also have contributed to observed changes in grade motivation. To address this limitation, future research will incorporate more comprehensive measures of academic context—such as examination-related stress, perceived academic stress, and recent academic performance—to more explicitly model the impact of contextual academic events on grade motivation. Fifth, regarding the basic developmental characteristics of the survey participants, this study only examined gender and did not include other potentially relevant factors, such as age. Future research should incorporate a broader range of demographic and developmental variables to enable a more comprehensive and precise analysis of the influencing factors. Furthermore, the measurement strategy may have introduced some limitations. Several scales were adapted, shortened, or newly translated for the purposes of this study. While reliability and overall model fit were acceptable, the loadings of some items were relatively low, which may have reduced the sensitivity of the measurement. Moreover, because only Metric invariance was established and Scalar invariance was not supported, the existing data are better suited for examining correlations across time than for drawing strong conclusions about changes in average levels. Finally, another limitation lies in the potential clustering effect at the school level. Although participants were not surveyed by complete class, they still belonged to four schools and may therefore share similar school environments. Because school identifiers were not preserved, we were unable to estimate multilevel models or calculate cluster robust standard errors. Consequently, the standard errors of regression coefficients may be underestimated, potentially leading to overly lenient significance tests. This issue is particularly significant given the small cross-lag coefficients that were statistically significant in this study. Therefore, these findings should be interpreted as preliminary and require replication in datasets that preserve school and class identifiers and allow for multilevel or cluster-adjusted analyses.

## 5. Conclusions

This study employed a two-wave cross-lag panel design to examine the prospective associations among intrinsic motivation, grade motivation, and prosocial behavior in Chinese adolescents. The results showed a positive correlation between intrinsic motivation at T1 and relational and practical prosocial behavior at T2, while no significant cross-lag associations were found between grade motivation and subsequent prosocial behavior, or between prosocial behavior and subsequent motivation. The findings of this study provide preliminary evidence for a prospective link between adolescents’ intrinsic motivation and prosocial behavior. However, given the methodological limitations of this study, including the two-wave design and potential clustering effects, these findings should be cautiously interpreted. Therefore, the conclusions should be considered suggestive rather than definitive.

## Figures and Tables

**Table 1:** Differences in the T1 variable between retained and unretained participants.

Variables	Retained M (SD)	Not Retained M (SD)	t	*p*	Cohen’s d
IM	3.448 (0.716)	3.444 (0.889)	0.068	0.939	0.01
GM	3.756 (0.675)	3.715 (0.736)	0.757	0.426	0.06
RB	3.504 (0.541)	3.526 (0.677)	−0.472	0.591	0.04
PB	3.640 (0.568)	3.719 (0.636)	−1.716	0.067	0.13

**Table 2:** Means, standard deviations, and paired-samples t-tests of main study variable.

Variables	M	SD	t	df	Sig (Two-Tailed)	Cohen’s d
T1 IM	3.448	0.716	2.728	861	0.006	0.093
T2 IM	3.381	0.718				
T1 GM	3.756	0.675	3.166	861	0.002	0.108
T2 GM	3.684	0.665				
T1 RPB	3.504	0.541	2.570	861	0.010	0.088
T2 RPB	3.459	0.507				
T1 PPB	3.640	0.568	3.011	861	0.003	0.103
T2 PPB	3.580	0.526				

M: mean, SD: standard deviation, IM: Intrinsic Motivation, GM: Grade Motivation, RPB: Relational Prosocial Behavior, PPB: Practical Prosocial Behavior, Cohen’s *d* refers to dz for paired samples (dz = t/√n).

**Table 3:** Pearson zero-order correlation matrix.

Variables	(1)	(2)	(3)	(4)	(5)	(6)	(7)	(8)
(1)T1 IM	1							
(2)T2 IM	0.502 **	1						
(3)T1 GM	0.198 **	0.139 **	1					
(4)T2 GM	0.149 **	0.261 **	0.500 **	1				
(5)T1 RPB	0.284 **	0.213 **	0.266 **	0.162 **	1			
(6)T2 RPB	0.232 **	0.313 **	0.172 **	0.303 **	0.522 **	1		
(7)T1 PPB	0.211 **	0.166 **	0.264 **	0.136 **	0.582 **	0.344 **	1	
(8)T2 PPB	0.167 **	0.279 **	0.171 **	0.309 **	0.369 **	0.644 **	0.446 **	1

** *p* < 0.01.

**Table 4:** Confirmatory factor analysis.

Variables	Item	Estimate	S.E.	Est./S.E.	*p*
T1 IM	IM1	0.810	0.019	42.147	0.000
	IM2	0.930	0.013	72.809	0.000
	IM3	0.782	0.025	31.625	0.000
T2 IM	IM1	0.791	0.022	35.874	0.000
	IM2	0.693	0.157	4.409	0.000
	IM3	0.798	0.022	36.917	0.000
T1 GM	GM1	0.894	0.024	37.745	0.000
	GM2	0.766	0.024	32.216	0.000
	GM3	0.587	0.028	20.724	0.000
T2 GM	GM1	0.929	0.021	43.528	0.000
	GM2	0.782	0.022	34.776	0.000
	GM3	0.622	0.028	22.245	0.000
T1 RPB	RPB1	0.717	0.041	17.543	0.000
	RPB2	0.491	0.038	12.950	0.000
	RPB3	0.642	0.035	18.453	0.000
	RPB4	0.656	0.032	20.591	0.000
T2 RPB	RPB1	0.781	0.029	26.489	0.000
	RPB2	0.496	0.044	11.359	0.000
	RPB3	0.665	0.033	20.224	0.000
	RPB4	0.688	0.036	19.085	0.000
T1 PPB	PPB1	0.812	0.023	35.190	0.000
	PPB2	0.764	0.023	32.537	0.000
	PPB3	0.463	0.033	13.814	0.000
	PPB4	0.563	0.029	19.388	0.000
T2 PPB	PPB1	0.773	0.025	31.032	0.000
	PPB2	0.720	0.025	28.759	0.000
	PPB3	0.415	0.034	12.309	0.000
	PPB4	0.512	0.032	16.199	0.000

**Table 5:** Invariance test.

	Model	χ^2^	df	RMSEA	CFI	TLI	SRMR	ΔRMSEA	ΔCFI
IM	Configural model	6.938	5	0.021	0.991	0.973	0.012		
	Metric invariance	10.955	7	0.026	0.982	0.961	0.025	0.005	−0.009
GM	Configural model	7.350	5	0.023	0.998	0.994	0.014		
	Metric invariance	8.790	7	0.017	0.998	0.997	0.020	−0.006	0.000
RPB	Configural model	24.575	15	0.027	0.991	0.984	0.021		
	Metric invariance	26.587	18	0.024	0.992	0.988	0.025	−0.003	0.001
PPB	Configural model	78.925	15	0.070	0.943	0.895	0.043		
	Metric invariance	83.644	18	0.065	0.942	0.910	0.050	−0.005	−0.001

## Data Availability

The data presented in this study are available on request from the corresponding author due to the research subjects are minors.
